# Improving Skills and Knowledge: Adapting a Core Competencies Suicide Risk Assessment Training Program to Support Mental Health Professionals in Hungary

**DOI:** 10.1192/j.eurpsy.2024.520

**Published:** 2024-08-27

**Authors:** M. Bérdi, R. J. Cramer

**Affiliations:** ^1^Psychiatry and Crisis Intervention, Peterfy Sandor Utcai Hospital-Clinic and Trauma Centre, Budapest, Hungary; ^2^Public Health Sciences, University of North Carolina at Charlotte, Charlotte, United States

## Abstract

**Introduction:**

Competency and skill-based education and assessment have become increasingly significant in mental health professional training. The conventional approach of acquiring knowledge is now being supplemented by emphasizing practical skills and implementing best practices that prove effective in the field. This emphasis on competencies is particularly apparent in the instruction regarding suicide risk evaluation and management. Cramer and colleagues have identified ten core competencies essential for working with patients at risk of suicide and developed a training material (Cramer et al. 2013, Train. Educ. Prof. Psychol; 1 1-11).

**Objectives:**

We aim to tailor Cramer et al.’s training program to the Hungarian setting and assess its efficacy among mental health experts, including psychiatrists, clinical psychologists, and social workers. Additionally, we aim to validate the Suicide Prevention and Assessment - Competency Assessment Form (SCAF-R), which comprises a ten-item survey to measure the ten core competencies’ levels with Likert scales and textual ratings by observers. Through this training program, we aim to offer mental health professionals an educational framework to enhance their skills in evaluating and managing suicide risk. Our goal is to provide a comprehensive approach to suicide risk assessment and better equip professionals to handle this emotionally difficult clinical task.

**Methods:**

We have created a Hungarian version of the core competencies training material tailored to the culture. We are assessing changes in attitudes towards suicide behavior and prevention by administering pre- and post-training psychometric measures, such as Willingness to Intervene against Suicide (WISE), Suicide Behavior Attitude Questionnaire (SBAQ), Attitudes Toward Suicide Prevention Scale (ASP), and Suicide Competency Assessment Form - Revised (SCAF-R). A quantitative analysis will be performed on the responses. The research was approved by Péterfy Hospital’s Institutional Review Board (IRB): approval number 07-2023.

**Results:**

The questionnaires’ results will be summarized with standard statistical methods.

**Conclusions:**

Improving mental health education in healthcare with up-to-date knowledge of evidence-based best practices is a top priority. Enhancing skills and knowledge can lower clinicians’ anxiety in this emotionally challenging and burdensome task. There is a high demand for mental health workshop training among healthcare workers in both undergraduate and post-graduate education. We expect positive changes in attitude and self-perceived competencies in participants.

**Disclosure of Interest:**

None Declared

